# Bioinformatics prediction and experimental validation of VH antibody fragment interacting with *Neisseria meningitidis *factor H binding protein

**DOI:** 10.22038/ijbms.2020.44007.10318

**Published:** 2020-08

**Authors:** Hediyeh Rafighdoust, Shahrzad Ahangarzadeh, Fatemeh Yarian, Ramezan Ali Taheri, Arezou Lari, Mojgan Bandehpour, Mona Salahshoor Dahr

**Affiliations:** 1 Department of Biology, School of Basic Sciences, Science and Research Branch, Islamic Azad University, Tehran, Iran; 2 Infectious Diseases and Tropical Medicine Research Center, Isfahan University of Medical Sciences, Isfahan, Iran; 3 Cellular & Molecular Biology Research Center Shahid Beheshti University of Medical Sciences, Tehran, Iran; 4 Department of Biotechnology, School of Advanced Technologies in Medicine Shahid Beheshti University of Medical Sciences, Tehran, Iran; 5 Nanobiotechnology Research Center, Baqiyatallah University of Medical Sciences,Tehran, Iran; 6 Systems Biomedicine, Pasteur Institute of Iran, Tehran, Iran; 7 Department of Biology, Faclty of Basic Science, Islamic Azad University Islamshahr Branch, Islamshahr, Iran

**Keywords:** Cloning, Heavy chain variable, Modeling, Molecular docking, Surface plasmon resonance

## Abstract

**Objective(s):**

We previously conducted an *in silico* research on the interactions between the ribosome display-selected single chain variable fragment (scFv) and factor H binding protein (fHbp) of *Neisseria meningitidis*. We found that heavy chain variable (VH) fragment of this scFv had considerable affinity to fHbp. These results led us to evaluate the ability of this small antibody fragment in binding and detection of fHbp antigen.

**Materials and Methods:**

In this study, at first, the three-dimensional structure of VH fragment was simulated by Kotai Antibody Builder web server. By using ClusPro 2.0 web server, the 3D structure of the soluble form of fHbp (PDB: 2KC0) was docked to the modeled VH fragment to extract the structure of the complex’s binding. Molecular dynamics (MD) simulation was carried out using GROMACS 4.5.3 package for 65 ns. Secondly, coding sequence of VH fragment was cloned separately and expressed in *Escherichia coli*. After purification of the VH fragment, its binding activity to fHbp protein was analyzed by enzyme-linked immunosorbent assay (ELISA) and surface plasmon resonance (SPR) method.

**Results:**

Important amino acids involved in antigen- antibody interaction were identified by analyzing the fHbp-VH complex. The ability of the VH antibody fragment to bind and detect fHbp antigen has been confirmed by the results of *in silico* analysis, ELISA and SPR methods.

**Conclusion:**

These results showed that this small fragment of antibody could be used for designing diagnostic kits.

## Introduction

Advances in bioinformatics and computational methods caused structure prediction of antibody and *in silico* study of the antigen-antibody interactions (1, 2). The most important web servers for antibody modeling are Kotai antibody Builder (https://bio.tools/kotai_antibody_builder) and PIGSpro (https://cassandra.med.uniroma1.it/pigspro/) that can build models of immunoglobulins by homology methods. In order to modeling of antibody structure, the antibodies’ crystal format was employed. This crystal structures are utilized as a template (3). In lack of the presence of experimental structures, protein- protein docking program is a good option to predict the conformation of protein complexes and binding interactions of antigens with antibodies (4).

Recently, a wide variety of antibody fragments provided possibilities for the production of new therapeutic and diagnostic agents. In comparison with the whole antibodies, these fragments have significant advantages including smaller size, simpler manufacturing processes, more efficient tissue penetration, and ability to generate multi-specific fragments. Genetic manipulations of these fragments are also feasible because of their small size (5). A single-chain fragment variable (scFv) is a polypeptide chain (~28 kDa). This polypeptide consisted of both heavy (VH) and light (VL) chain variable regions of an immunoglobulin. There is also a short polypeptide (10-25 amino acids) linker, which has made a covalently link. Because of glycine and serine residues in linker structure, scFv would be remained flexible and resistant to proteases (6).

In our previous studies, we identified and characterized a scFv antibody against *Neisseria meningitidis* factor H binding protein (fHbp) (7). Bioinformatics evaluation of the interactions between the anti-fHbp scFv and fHbp showed that VH fragment of this scFv had considerable affinity to fHbp. Docking led to identifying the essential interacting residues in both VH and VL chains regions of scFv (8). 

 In this study, VH fragment of the selected anti-fHbp scFv was separately cloned and expressed in *Escherichia coli*. After purification of the VH fragment coding sequence, its binding activity to fHbp protein was analyzed utilizing enzyme-linked immunosorbent assay (ELISA) and surface plasmon resonance (SPR) methods.

## Materials and Methods

***Modeling of VH antibody fragment and fHbp protein***

In order to generate the three-dimensional (3D) structure of the VH fragment, Kotai Antibody Builder (PDB: 1jhl) was employed (9). This web server builds 3D structures of variable domains of antibodies using their sequence. Modeller 9.16 has been utilized to construct the model of fHbp antigen. The 3D structure of the soluble form of fHbp (PDB: 2KC0) was applied as a scheme for fHbp protein homology modeling. After modeling of VH fragment and fHbp protein, the energy minimization process was made using steepest descent algorithm of GROMACS 4.5.3 package.

***Molecular docking of VH antibody fragments and fHbp protein and molecular dynamics simulation***

Antibody-antigen molecular docking was performed by ClusPro 2.0 (https://cluspro.bu.edu/publications.php) while working in antibody mode. Identifying the antibody and antigen was carried out by considering antibody and antigen as receptor and ligand, respectively. All default parameters were accepted during the analysis. Automatic masking of non-Complementarity-determining regions (CDRs) regions of the antibody chains was utilized to obtain better docking results. In order to analyze, the models that have the lowest energy level were chosen as the best models (10). Furthermore, for molecular dynamics (MD) simulation, the finest docked model constructed by ClusPro was employed. Then, the energy of the whole molecular system was minimized, while the number of iterations was 5000 and the steepest descent algorithm was applied to implement GROMACS96 43a1 force field. 

GROMACS 4.5.3 package was employed to simulate the complexes of antibody-antigen. In order to adjust the inside box temperature, we used Berendsen temperature coupling method. The procedure that was used for computing electrostatic interactions was Particle Mesh Ewald technique. The pressure was preserved at 1 atm and the permissible compressibility range is 4:5×10^-5^ atm. The linear constraint solver algorithm was worked to constrain bond lengths involving hydrogen atoms, permitting a time step of 2 fsec (11), while Van der Waals and coulomb interactions were truncated at 1.0 nm. Updating the non-bonded pair list and confirmation were performed every 10 steps and 0.5 psec, respectively. The subsequent system was subjected to MD simulation for 30 nsec. Then, root mean square deviation (RMSD), radius of gyration (Rg), and the number of hydrogen bonds between VH antibody fragment-fHbp complexes were achieved to predict dynamic behavior and structural changes of the studied VH antibody fragment.

***Cloning of VH antibody fragment into pET28a (+) expression vector***

DNA sequence of the VH fragment antibody (7) was amplified using VH/back (5´ NcoI *CCATGG *(C/G)AGGT(G/C)CA(G/C)CTCGAG(C/G)AGTCTGG3΄ and VH/for (5´NotI* GCGGCCGC*TG AGGAGACGGTGACCGTGGTCCCTTGGCCCC3´) primers by PCR (94 ^°^C for 40 sec, 54 ^°^C for 1 min and 72 ^°^C for 1 min, 30 cycles) from the DNA coding the selected scFv antibody (7).

The 351 bp fragment of VH PCR product and pET28a (+) (Novagen, USA) were digested with NcoI and NotI (ThermoFisher Scientific, USA). After agarose gel extraction (AccuPrep^®^ Gel Purification Kit, Bioneer), ligation was carried out with T4 DNA Ligase (ThermoFisher Scientific, USA), and the ligation product was transformed into E. coli Top10 competent cells. The recombinant plasmids were confirmed by colony PCR with universal primers of pET28a (+), restriction enzyme analysis, and Sanger sequencing.

***Expression and purification of VH antibody fragment***

*E. coli *BL21 (DE3) was transformed with pET28a-VH antibody fragment. It was grown in 50 ml of LB medium containing 50 μg/ml Kanamycin at 37 ^°^C with shaking at 180 RPM to a density of OD_600_=0.6. Then, Isopropyl-D-1-thiogalactopyranoside (IPTG) (Merck, Germany) was added to a final concentration of 1 mM. Samples were collected at 3, 5 and 8 hr after induction and treated with lysis buffer (Tris 50 mM, 10% glycerol, 0.1% Triton X-100) (Merck, Germany). The cell lysate was evaluated by SDS-PAGE (12).

***ELISA-based evaluation of the binding activity of VH antibody fragment***

Microtiter plate was coated with recombinant fHbp protein (2 μg/ml in phosphate buffer (PBS), pH=7.5) and incubated 16 hr at room temperature. PBS containing 3% (w/v) bovine serum albumin (BSA) was used for blocking for 2 hr. After three washing steps, 100 μl of extracted VH fragment diluted with PBST (5 μg/ ml) was added to the wells as the primary antibody and incubated for 7 hr at 37 ^°^C. Horseradish peroxidase (HRP)-conjugated anti-His tag antibody (2 μg/l in PBS+0.05% Tween 20) (Abcam, UK) was used as the secondary antibody. The immunoreaction was started by the addition of 100 μl of TMB (tetramethylbenzidine) (13). After 15 min, the peroxidase reaction was stopped by adding 100 μl of 2N H_2_SO_4_. Absorbance was measured at 450 nm. Finally, the affinity of VH fragment against recombinant fHbp antigen was determined by ELISA. Affinity constant (K_aff_) was calculated using the method described by Beatty *et al*., (14). Different concentrations (2, 1, 0.5, 0.25 and 0.125 μg/ml) of purified recombinant fHbp in PBS buffer (pH=7.5) were used to coat the microplate wells. The microplate was incubated at 16 hr at room temperature. For each concentration of fHbp protein, 5, 2.5, 1.25, and 0.625 μg/ml of purified VH fragment were used separately. The Beatty’s equation was used to evaluate K_aff_ of VH fragment and fHbp antigen. 

***Surface Plasmon Resonance analysis of VH fragment antibody and fHbp protein interactions***

*Apparatus, chemicals, and reagents*

A double channel cuvette-based Surface Plasmon Resonance (SPR) (Autolab ESPRIT, Ecochemie B.V., Netherlands) was used for analysis of interaction between VH fragment and fHbp protein and calculation of the affinity of interaction. One channel was used for the test, and the other was used for reference sample measurements. The result of the SPR measurement was automatically monitored using data acquisition software version 4.3.1. All kinetic data were achieved using the SPR kinetic evaluation software version 5 (Ecochemie B.V.). 

11-Mercaptoundecanoic acid (11-MUA), 1-ethey 3-3 dimethyiaminopropyl carbodiimide hydrochloride (EDC), N-hydroxysuccinimic (NHS), HEPES (4-(2-hydroxyethyl)-1-piperazineethanesulfonic acid) and Ethanolamine were purchased from Sigma-Aldrich (Steinheim, Germany). 

***Antibody immobilization on gold disc***

In this study, VH fragment antibody was covalently immobilized on 11-MUA linker via amine coupling. Amine coupling is the most generally applicable covalent coupling chemistry used to immobilize protein ligands (15). For immobilization of VH fragment, at first, the surface of gold disks was cleaned using piranha solution (3:1 v/v mixture of 98% H2SO4:30% H2O2). In order to form self-assembled monolayer on gold disk, the gold disc was immersed in 11-MUA solution (0.001 mol l^-1^) overnight. Second, the surface of MUA/gold disk was washed with acetate buffer (0.01 mol l^-1^) for immobilization of antibody on the gold disk. At this point, resonance angle was recorded as baseline. Then, MUA/gold disk was gained by injecting 100 μl of freshly prepared 1:1 mixture of EDC (0.4 mol l^-1^) and NHS (0.1 mol l^-1^) in distilled water (DW) over the MUA/gold disk for 300 sec. Afterwards, 50 μl of VH antibody fragment (60 μg ml^-1^) was immobilized on MUA/gold disk surface. After 800 sec, it was washed by coupling buffer (NaAc.3H_2_O). After immobilization of VH antibody fragment on MUA/gold disk, neutralizing unreacted activated ester groups on the formed 11-MUA with 1 mol l^-1^ ethanolamine (pH = 8.5) is crucial. Channel 1 was utilized to immobilize the VH antibody fragment, whereas channel 2 was employed as negative control without VH.

VH fragment was coated on MUA/gold disk surface. Then, different concentrations of fHBP (5, 2.5, 1.25, 0.625, 0.3125, and 0.156 μg ml^-1^) were passed over the disk, and the sensograms in optimized pH of coupling buffer (NaAc.3H_2_O) were achieved (16).

## Results

***Homology modeling, molecular docking and MD simulation***

**Figure 1:**
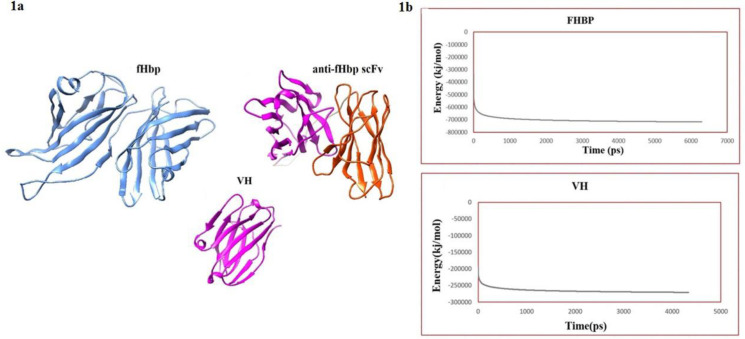
a) Three-dimensional models of fHbp, anti-fHbp scFv, and VH antibody fragment. b) Energy minimization of fHbp and VH antibody fragment. Minimization stopped when the maximum force < 100 kj/mol/nm

Using homology modeling method by Modeller 9.16, three-dimensional model of fHbp antigen was built (Figure 1a). The 3D models of the scFv antibody and VH fragment antibody were created using Kotai Antibody Builder (Figure 1a). The energy minimization process of both models was made using steepest descent algorithm of GROMACS package (Figure 1b).

**Figure 2:**
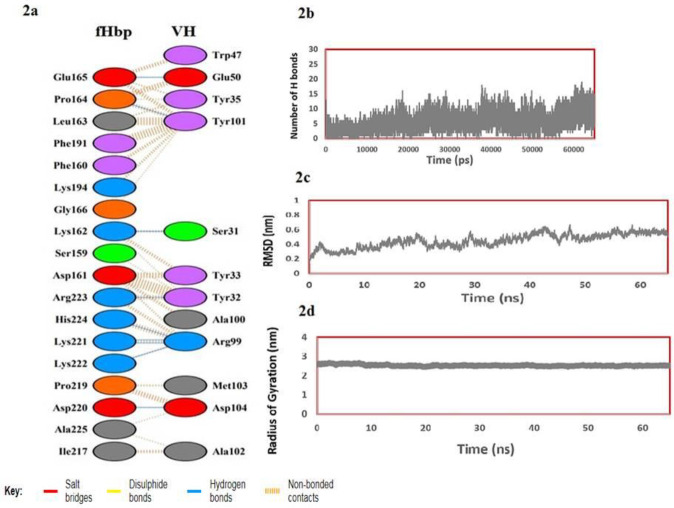
a) Amino acids involved in the antigen– heavy chain (VH) fragment complex. Residues of heavy chain of antibodies associated with the factor H binding protein (fHbp) are shown. b) The number of hydrogen bonds between VH antibody fragment and fHbp antigen. c) Root mean square deviation (RMSD) evolution for all backbone atoms of Antigen-Antibody complex. d) Radius of gyration (Rg) of Antigen-Antibody complex

Molecular docking between fHbp and the VH fragment antibody was considered using ClusPro 2.0 to study the binding mechanism and interaction modes of VH fragment antibody (Figure 2a). Among the 10 models built by the ClusPro 2.0 server, the greatest model with the lowest energy was selected. The docked complex was then simulated using GROMACS 4.5.3 package (11). Protein system was solvated in a triclinic box with simple point charge water model. The structure was found to be positively charged at pH=7.4. Therefore, Chloride ion (Cl^-^) was added to the simulation box to create the system electrically neutral. Electrostatic interactions were calculated using Particle Mesh Ewald method (PME) (17). The pressure was maintained at 1.0 atm. Then, the system was subjected to MD simulation for 65 nsec. Figure 2b shows the number of hydrogen bonds between VH antibody fragment and fHbp antigen during simulation time. Root Mean Square Deviation (RMSD) analysis is presented in Figure 2c. After 3 nano second, structure of the protein reaches a certain distance from the reference structure and then keeps that distance more or less, until it reached plateu on 54 nsec and maintain its value.

***Cloning, expression, and purification of VH fragment antibody***

**Figure 3:**
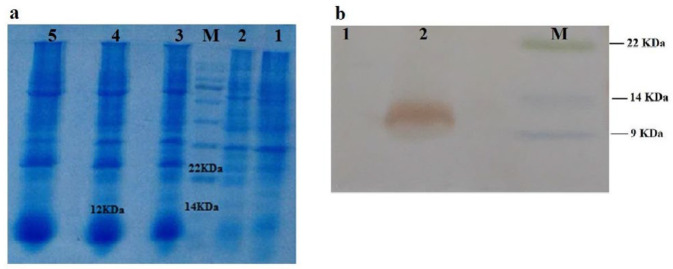
SDS-PAGE (a) and Western blot (b) analysis of heavy chain (VH) fragment expression after induction. (a) SDS-PAGE analysis following coomassie brilliant blue G250 staining. Lane 1, BL21 cell lysate; Lane 2, t0; lane M, protein molecular mass marker ; Lane 3, 3 hr; Lane 4, 5 hr and Lane 5, 7 hr after induction with 1 mM isopropyl-D-1-thiogalactopyranoside (IPTG). (b) Western blot analysis using anti his-tagged HRP antibody. lane M, protein molecular mass marker, Lane 1, BL21 cell lysate, Lane 2, VH fragment expression after induction with IPTG (12 kDa)

The sequences of VH fragment was amplified with VH/for-NcoI and VH/back NotI primers. The PCR product was ligated into pET28a (+) expression vector. The His-tagged VH fragment antibody was expressed in *E. coli *BL21 (DE3) and purified by Ni-NTA agarose resin. The expressed protein (12 kDa) was investigated by SDS-PAGE and western blotting (Figure 3). 

***Functional assay of the purified scFv by ELISA***

**Figure 4:**
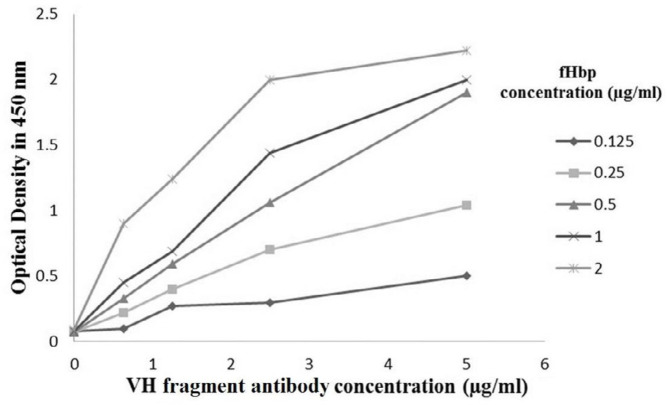
Affinity of heavy chain (VH) fragment antibody was measured by ELISA. The affinity constant (K_aff_) was 2.25×10^10^ M^-1^

Affinity constant (K_aff_) of the purified VH fragment was determined to be 2.25×10^10^ M^−1^ (K_aff_ is the antigen-antibody affinity constant in l/mol (M^-1^)), (Figure 4).

***Affinity investigation of antigen on VH/ MUA/gold disk by SPR***

**Figure 5:**
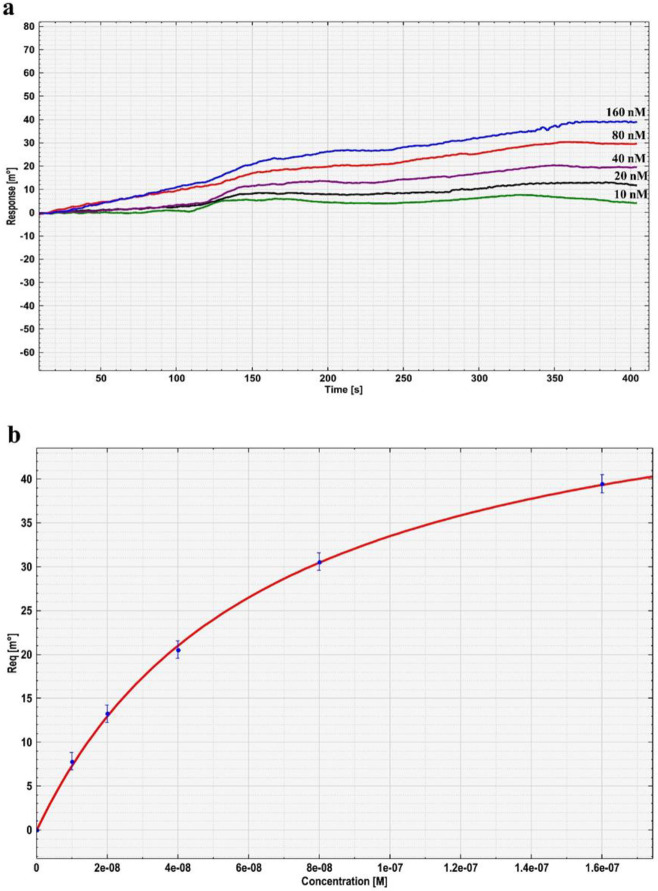
Interaction of immobilized heavy chain (VH) fragment antibody (60 μg ml) with different concentration of factor H binding protein (fHbp). (A) Surface Plasmon Resonance (SPR) sensor response overlay plot for the interaction of various concentrations (10–160 nM) of fHbp recombinant antigen with 60 μg/ml immobilized VH antibody fragment (B) Langmuir isotherm plot of equilibrium angle (Req) versus fHbp protein concentration

For the assessment of VH affinity, firstly, various concentrations of the antigen (fHbp) from 9×10^-9^ to 3×10^-7^ mol l^-1^ was passed over the VH fragment antibody/MUA/gold disk surface. Antibody-antigen interaction graph is completely evident. Thus, VH/MUA/gold disk has a suitable affinity against fHbp. Affinity constant was determined to be 9.74×10^9^ M^−1 ^(Figure 5).

fHbp: factor H binding protein, scFv: single chain variable fragment, VH: heavy chain

## Discussion

In the treatment of infectious diseases, rapid, accurate, and precise detection of pathogens is very important.Detection of pathogenic bacteria can be performed by using antibodies.

There are different methods to produce new generation of diagnostic and therapeutic agents, thanks to innovative technologies such as antibody fragments technology and recombinant DNA advances. Recombinant antibody fragments (rAbFs) are minimal antigen-binding proteins with the full antigen-binding capacity of whole antibodies. It has been shown that scFv, Fab (fragment antigen binding), and Nanobodies (Nbs) have the advantages of economical production, superior biodistribution, easy genetic manipulation, and chemical modification. These advantages enable us to generate fragments with desired properties. The development of selection technologies (e.g. phage- and ribosome-display) and the advent of various production systems have simplified rAbF production. 

*Meningococcal meningitis *is a serious bacterial infection caused by the gram-negative bacterium *N. meningitidis*. This infection causes inflammation and swelling of the membranes that cover brain and spinal cord and can be fatal if it is not treated quickly and efficiently. fHbp, a surface-exposed lipoprotein of *N. meningitidis*, is the main virulence factor. fHbp is used in vaccines since it is expressed by all strains of serogroup B meningococcus (18). This antigen binds to factor H (fH), a regulatory protein of the complement system, and recruits fH ‌ to the surface of the bacterium. Interaction and binding of fH with fHbp down-regulates alternative complement pathway. Thus, the bacterium evades host innate immunity and survives in human serum (19, 20).

Although, crystal experiment is one of the best methods for studying protein-ligand binding mechanism, it is time-consuming. Computational biology can solve the problem. It is a conceivable method to study the protein-ligand interaction on *in silico* at a suitable speed.

Payandeh *et al.*, designed new antibody using computational biology and *in silico* approach by rational engineering methods (21).

In this research, according to our previous *in silico* study (22), a new bioinformatics study was performed on the VH fragment and fHbp antigen. In our selected scFv, the number of amino acids involved in antibody’s heavy chain and antigen interaction was more than the light chain. The VH fragment was successfully cloned in the pET28a vector and expressed in *E. coli *BL21 (DE3)*.* After extraction and purification of the expressed VH, ELISA assay was used for initial analysis of the interaction between VH and fHbp. Affinity constant was 2.25×10^10^ M^−1^ for VH. This is 2.5 times more than the affinity of complete scFv (8.65× 10^9^ M^−1^) (7).

SPR binding analysis was performed to further investigate the affinity of this VH antibody fragment. SPR-based optical biosensor is a powerful technology for detecting the molecular interactions of two different molecules one of which is mobile and one is fixed on a thin nanolayer gold film (50 nm). The affinity calculated by this method was 9.74× 10^9^ M^−1^. The reason for the difference between affinities obtained by ELISA and SPR may be due to the difference in the basis of these methods. In ELISA, the antigen is coated on microtiter plates, and VH fragment is added to the plates, but in SPR the antibody is fixed on a disk. Therefore, the VH fragment in ELISA is more flexible than the VH fragment coated on disks. 

Nowadays, using *in silico*-based methods, molecular interactions between antigen and antibody fragments can be predicted, and the results can be used to design and select antibodies with the highest affinity. On the other hand, the affinity of selected antibodies can be evaluated by both ELISA and SPR methods.

## Conclusion

In the present study, the results of bioinformatics studies, ELISA, and SPR confirmed the ability of the VH antibody fragment to bind and detect fHbp antigen. These results showed that this small fragment of antibody could be used in designing diagnostic kits.
